# Length of urban residence and obesity among within-country rural-to-urban Andean migrants

**DOI:** 10.1017/S1368980015002578

**Published:** 2015-09-14

**Authors:** Daniel A Antiporta, Liam Smeeth, Robert H Gilman, J Jaime Miranda

**Affiliations:** 1 CRONICAS Center of Excellence in Chronic Diseases, Universidad Peruana Cayetano Heredia, Av. Armendáriz 497, Miraflores, Lima 18, Peru; 2 Faculty of Epidemiology and Population Health, London School of Hygiene and Tropical Medicine, London, UK; 3 Asociación Benéfica PRISMA, Lima, Peru; 4 Program in Global Disease Epidemiology and Control, Department of International Health, Bloomberg School of Public Health, Johns Hopkins University, Baltimore, MD, USA; 5 School of Medicine, Universidad Peruana Cayetano Heredia, Lima, Peru

**Keywords:** Obesity, Migration, Rural-to-urban, Peru, Skinfold, Nutritional epidemiology

## Abstract

**Objective:**

To evaluate the association between length of residence in an urban area and obesity among Peruvian rural-to-urban migrants.

**Design:**

Cross-sectional database analysis of the migrant group from the PERU MIGRANT Study (2007). Exposure was length of urban residence, analysed as both a continuous (10-year units) and a categorical variable. Four skinfold site measurements (biceps, triceps, subscapular and suprailiac) were used to calculate body fat percentage and obesity (body fat percentage >25% males, >33% females). We used Poisson generalized linear models to estimate adjusted prevalence ratios and 95 % confidence intervals. Multicollinearity between age and length of urban residence was assessed using conditional numbers and correlation tests.

**Setting:**

A peri-urban shantytown in the south of Lima, Peru.

**Subjects:**

Rural-to-urban migrants (*n* 526) living in Lima.

**Results:**

Multivariable analyses showed that for each 10-year unit increase in residence in an urban area, rural-to-urban migrants had, on average, a 12 % (95 % CI 6, 18 %) higher prevalence of obesity. This association was also present when length of urban residence was analysed in categories. Sensitivity analyses, conducted with non-migrant groups, showed no evidence of an association between 10-year age units and obesity in rural (*P*=0·159) or urban populations (*P*=0·078). High correlation and a large conditional number between age and length of urban residence were found, suggesting a strong collinearity between both variables.

**Conclusions:**

Longer lengths of urban residence are related to increased obesity in rural-to-urban migrant populations; therefore, interventions to prevent obesity in urban areas may benefit from targeting migrant groups.

Overweight and obesity currently affect more than 50 % of the female population in Peru^(^ ^1^ ^)^, a country undergoing an epidemiological and nutritional transition, especially in urban areas^(^ ^2^ ^–^ ^4^ ^)^. This transition has not only affected the urban population but also the rural-to-urban migrant population with residence in peri-urban areas of Peru^(^ ^5^ ^)^. The living conditions facing many rural-to-urban migrants, including poverty, restricted access to health care^(^ ^6^ ^–^ ^8^ ^)^ and the acculturation process, can increase their chances to develop obesity, diabetes and other non-communicable diseases compared with non-migrants^(^ ^9^ ^,^ ^10^ ^)^. Different techniques other than BMI, such as bioelectrical impedance, waist-to-hip ratio and skinfold measurements, provide a more detailed assessment of the excess of body fat mass^(^ ^11^ ^–^ ^13^ ^)^.

Previous studies that measured the effect of the length of urban residence among migrants and the risk of obesity have shown conflicting results. Some studies demonstrated a significant positive effect^(^ ^14^ ^,^ ^15^ ^)^ whereas others did not^(^ ^16^ ^,^ ^17^ ^)^. One possible explanation for these conflicting data is the potential multicollinearity existing between length of residence in an urban area, age at first migration and age, which has not been properly explored^(^ ^18^ ^,^ ^19^ ^)^.

Using skinfold measurements, we assessed the relationship between the length of residence in an urban area and obesity in rural-to-urban migrants from the PERU MIGRANT Study^(^ ^20^ ^)^, including the examination of multicollinearity between three time-related factors: length of urban residence, age of first migration and age.

## Methods

### Study design

The present study is a cross-sectional database analysis of the PERU MIGRANT Study. The PERU MIGRANT Study was a population-based, age- and sex-stratified cross-sectional study with the objective of characterizing differences in cardiovascular risk profiles in rural, rural-to-urban migrant and urban groups. Details and main findings of the PERU MIGRANT Study have been published elsewhere^(^ ^5^ ^,^ ^20^ ^)^.

### Participants

All participants in the PERU MIGRANT Study were ≥30 years old. For the main analysis we only included data from rural-to-urban migrants: people born in an Andean rural area, San José de Secce in Ayacucho, who migrated to urban areas and are currently living in a shantytown called Papas de San Juan de Miraflores in Lima, Peru’s capital city located in the coastal region. To ensure consistency with age intervals in the Durnin and Womersley equation for the assessment of body fat percentage^(^ ^21^ ^)^, we excluded males aged >72 years (*n* 19) and females aged >68 years (*n* 32).

### Variables of interest

The exposure, length of residence in urban areas, was assessed in the migrant population by the question ‘On average, how many years have you lived in an urban setting?’ First, we used the variable as a scaled continuous variable where one unit was equal to 10 years of urban residence. We also categorized this variable into four groups: <20 years, 20–29 years, 30–39 years and ≥40 years.

Obesity was calculated using the sum of four skinfold sites: biceps, triceps, subscapular and suprailiac. Evaluators were health professionals trained in anthropometric measurements using skinfolds; they were standardized using the kappa statistic (*κ*≥0·8). Each skinfold site was measured in triplicate to the nearest 0·2 mm using a Holtain Tanner/Whitehouse Skinfold Caliper; the average of those measurements was recorded as the final result. The Durnin and Womersley^(^ ^21^ ^)^ equation was used to calculate specific body density by age and sex, and the Siri specific equation was used to calculate body fat percentage^(^ ^22^ ^)^. The cut-off points used for the classification of obesity, our outcome of interest, were established by the Spanish Society for Obesity Studies^(^ ^23^ ^)^ and were sex-specific: >25 % for males and >33 % for females^(^ ^23^ ^)^. In addition to skinfolds and given the familiarity with BMI categories, we also considered overweight (BMI=25·0–29·99 kg/m^2^) and obesity (BMI≥30·0 kg/m^2^) as secondary outcomes.

Other variables of interest included were age, sex and socio-economic factors, the latter being assessed through education level and, separately, using a deprivation index that aggregated education level, household income, number of people per room and asset possession^(^ ^24^ ^)^. Additionally, to control for the possible effects of acculturation to a Western lifestyle^(^ ^18^ ^)^ on body fat mass, we also adjusted for self-reported current smoking status (yes, no), alcohol drinking (never, ≤1 time/month and ≥2 times/month) and physical activity (low, moderate and high level using individual MET scores, where MET=metabolic equivalents of task). Details on the generation and aggregation of these variables are reported in previous PERU MIGRANT Study publications^(^ ^5^ ^,^ ^20^ ^)^.

### Statistical analysis

The association between length of urban residence, both as a continuous and a categorical exposure, and obesity was assessed by Poisson generalized linear models with robust variance to calculate prevalence ratios (PR) and 95 % confidence intervals controlling for potential confounding factors^(^ ^25^ ^)^.

We conducted the analyses using two different models: (i) Model A included length of urban residence adjusted by sex and age at first migration; (ii) Model B adjusted for sex, age at first migration, deprivation index, education level, physical activity, smoking status and alcohol consumption. In the analysis of the exposure as a categorical variable, both models used the <20 years of length of urban residence as the reference group. These analyses were repeated for the secondary outcomes based on BMI categories using a multinomial logistic regression to allow comparisons between overweight and obesity against the normal category as the base outcome; thus relative prevalence ratios were calculated for each category of BMI except for the underweight population (*n* 3) which was excluded from latter analyses.

Correlation between length of urban residence (exposure) and age, as well as age and the sum of age at first migration and length of urban residence, was explored using Spearman tests. To explore multicollinearity between length of urban residence and time-related variables, we also calculated an additional model including length of urban residence, age at first migration and current age. This was done because in the case of rural-to-urban migrant populations the age of an individual, in most cases, corresponds to the sum of age at first migration and time in urban areas^(^ ^18^ ^)^.

To avoid over-adjustment and the introduction of collinearity with age in our associations of interest, we explored multicollinearity using post-regression analysis (Model C). We conducted a post-regression diagnosis adding age into Model B using the variance inflation factor (VIF), the correlation matrix of coefficients and the independence coefficient matrix^(^ ^26^ ^,^ ^27^ ^)^. Conditional numbers derived from the matrix of independent variables greater than 30 indicate serious problems of multicollinearity in the regression models^(^ ^26^ ^)^, as do VIF values greater than 10^(^ ^28^ ^)^.

For comparison purposes, and given the time-dependent nature of our association of interest, a sensitivity analysis was conducted in non-migrant groups to explore the effect of age, as a continuous variable in 10-year units, on obesity using the same regression equations as in Model B by replacing age at first migration and length of urban residence for age.

All analyses were conducted using the statistical software package STATA version 12 for Windows.

## Results

### Participants and characteristics of the study population

We included 526 rural-to-urban migrants in the analysis, 52·3 % female, mean age 46·1 (sd 9·87) years (range 30–71 years), mean age of first migration 14 (sd 6·91) years (range 0–50 years), mean length of residence in urban areas 31·5 (sd 9·52) years (range 7–58 years). The overall prevalence of obesity according to the Spanish Society for Obesity Studies was 78 % (*n* 412). Table 1 shows the different sociodemographic characteristics of the rural-to-urban migrant population, including missing data in each category.

**Table 1:** Sociodemographic characteristics of rural-to-urban migrants according to obesity as assessed by skinfolds, PERU MIGRANT Study, 2007

	Rural-to-urban migrants (*n* 526)					
	Obese		Non-obese			
	Mean or *n*/*N*	sd or %	Mean or *n*/*N*	sd or %	*P* value†	Missing values
Length of urban residence (continuous, 10-year units), mean and sd	3·22	0·93	2·90	0·99	<0·05‡	–
Length of urban residence (categories)						–
<20 years	33/49	67·4	16/49	32·6		
20–29 years	143/197	72·6	54/197	27·4		
30–39 years	136/160	85·0	24/160	15·0		
≥40 years	100/120	83·3	20/120	16·7	<0·05	
Sex						–
Female	253/275	92·0	25/275	8·0		
Male	159/251	63·4	92/251	36·6	<0·001	
Age (years), mean and sd	46·92	9·87	43·06	10·59	<0·001‡	–
Age at first migration (years), mean and sd	14·18	6·89	13·47	6·96	0·33‡	–
Education level						1
None/some primary	127/145	87·6	18/145	12·4		
Primary complete	65/85	76·5	20/85	23·5		
Some secondary or more	219/295	74·2	76/295	25·8	<0·05	
Deprived household*						–
Yes	64/83	77·1	19/83	22·9		
No	348/443	77·6	95/443	22·4	0·77	
Physical activity						6
Low	119/148	80·4	29/148	19·6		
Moderate	158/192	82·3	34/192	17·7		
High	132/180	73·3	48/180	26·7	0·09	
Drink alcohol (in the last year)						16
Never	71/84	84·5	13/84	15·5		
≤1 time/month	296/383	77·3	87/383	22·7		
>2 times/month	33/43	76·7	10/43	23·3	0·33	
Currently smoke						–
Yes	37/55	67·3	18/55	32·7		
No	375/471	79·6	96/471	20·4	0·04	
BMI						–
Underweight (<18·50 kg/m^2^)	0/3	0·0	3/3	100·0		
Normal (18·50–24·99 kg/m^2^)	83/167	49·7	84/167	50·3		
Overweight (25·0–29·99 kg/m^2^)	218/244	89·3	26/244	10·7		
Obese (≥30·0 kg/m^2^)	111/112	99·1	1/112	0·9	<0·001	

* Deprived household was assessed by the deprivation index, an index that includes education level, household income, the number of people per room and asset possession.

† *P* values determined by *χ* ^2^ tests.

‡ *P* values determined by *t* test of means.

### Urban residence and obesity

Migrant groups with longer time of urban residence showed a higher prevalence of obesity than the reference group (*P* for trend=0·001), and it was shown predominantly in the female population (*P*<0·001). On the bivariate analysis, there was evidence of an association between obesity and age, education level and smoking status, but not with physical activity, deprivation index or alcohol consumption (Table 1).

Multivariable Poisson linear analyses showed that for each increase in 10-year unit of residence in an urban area, rural-to-urban migrants had 12 % higher prevalence of obesity (Table 2).

**Table 2:** Prevalence ratios and adjusted prevalence ratios for the association between length of residence in urban area and obesity as assessed by skinfolds, PERU MIGRANT Study, 2007

	Crude model		Model A		Model B	
	Unadjusted PR	95 % CI	Adjusted PR	95 % CI	Adjusted PR	95 % CI
Length of urban residence (continuous, 10-year units)	1·08	1·03, 1·13	1·1	1·05, 1·16	1·12	1·06, 1·18
Length of urban residence (categories)						
<20 years	1·00	Ref.	1·00	Ref.	1·00	Ref.
20–29 years	1·08	0·87, 1·33	1·13	0·94, 1·37	1·17	0·96, 1·42
30–39 years	1·26	1·03, 1·55	1·36	1·14, 1·69	1·39	1·14, 1·69
≥40 years	1·24	1·00, 1·53	1·33	1·12, 1·71	1·38	1·12, 1·71

PR, prevalence ratio; Ref. reference category. Model A shows adjusted PR from the multivariable Poisson generalized linear model that included sex and age at first migration. Model B is equal to Model A adjusted also by deprivation index, education level, smoking status, physical activity and alcohol consumption.

When analysed in categories of duration of residence in urban areas, and compared with the <20 years reference group, the groups with 30–39 years and ≥40 years of urban residency had consistently higher prevalence of obesity. This association became stronger with further adjustment, from 26 % higher in the crude model to 39 % in the fully adjusted model (Model B) for the group with 30–39 years of urban residency. This pattern was not observed in the category of 20–29 years of urban residency (Table 2).

Sensitivity analyses conducted in non-migrant groups showed no evidence of an association between age and obesity in rural (*P*=0·159) or urban groups (*P*=0·078). Data from a total of 184 rural and 182 urban participants were analysed. For each 10-year increase in age, PR estimates were 1·18 (95 % CI 0·90, 1·54) in the rural group and 1·05 (95 % CI 0·99, 1·10) in the urban group (data not shown).

### Multicollinearity evaluation

Correlation between age and length of urban residence was suggested by the graph matrix (see online supplementary material, Supplemental Fig. 1) and was confirmed with Spearman’s tests between length of urban residence and age (*r*=0·73), as well as age and the sum of length of residence and age at first migration (*r*=0·96).

We also analysed the effects of age in the association of interest. Adding age to the models weakened all the estimates, and all of the associations between length of urban residence and the obesity, as described before, became non-significant (see online supplementary material, Supplemental Table 1, Model C). The correlation matrix of coefficients resulted in a high rho coefficient (0·87) and a large conditional number shown in the matrix of independent variables (44·41) strongly linked with age (0·99) and length of urban residence (0·93). Furthermore, the mean VIF for Model C was 33·45; age (VIF=196·5) and length of urban residence (VIF=98·9) VIF values suggested a high multicollinearity effect.

Our evaluation of multicollinearity using post-regression diagnosis such as the VIF, correlation matrix and conditional numbers reinforced the approach followed in Model B and the estimates obtained from it as our main findings.

### Secondary outcomes by BMI categories

Obese participants, as per skinfolds, had a higher mean BMI than non-obese participants (28·1 *v*. 23·1 kg/m^2^, *P*<0·001); the kappa estimate showed moderate agreement between obesity by skinfolds and BMI (*κ*=42·59 %, *P*<0·001). BMI categories, shown in Table 1, revealed that 99·1 % of participants classified as obese by BMI, were classified as obese by the methodology used in our study. Also, half of those in the normal BMI category were deemed obese by skinfolds definition.

Table 3 displays results from multinomial regression analysis by BMI categories, using the normal category as the base outcome. Multivariable analysis of length of urban residence as continuous 10-year units showed no association in both overweight and obesity outcomes, as demonstrated by estimates spanning the value of 1. Whereas no evidence of a difference was displayed in prevalence of overweight among length of urban residence categories, obesity prevalence among categories differed and was greater than 1 shown in the reference group (<20 years).

**Table 3:** Prevalence ratios and adjusted prevalence ratios for the associations between length of residence in urban area and overweight and obesity as assessed by BMI, PERU MIGRANT Study, 2007

	Crude Model		Model A		Model B	
	Unadjusted PR	95 % CI	Adjusted PR	95 % CI	Adjusted PR	95 % CI
Overweight						
Length of urban residence (continuous, 10-year units)	0·96	0·78, 1·18	1·03	0·83, 1·28	0·99	0·78, 1·26
Length of urban residence (categories)						
<20 years	1·00	Ref.	1·00	Ref.	1·00	Ref.
20–29 years	1·37	0·71, 2·69	1·61	0·81, 3·22	1·61	0·77, 3·33
30–39 years	1·41	0·70, 2·81	1·75	0·86, 3·60	1·52	0·70, 3·27
≥40 years	1·13	0·55, 2·31	1·46	0·69, 3·09	1·39	0·61, 3·18
Obesity						
Length of urban residence (continuous, 10-year units)	1·09	0·84, 1·41	1·26	0·96, 1·65	1·19	0·88, 1·61
Length of urban residence (categories)						
<20 years	1·00	Ref.	1·00	Ref.	1·00	Ref.
20–29 years	**3**·**91**	**1**·**25**, **12**·**22**	**5·76**	**1**·**76**, **18**·**86**	**5**·**59**	**1**·**64**, **18**·**99**
30–39 years	**4**·**56**	**1**·**45**, **14**·**42**	**7·77**	**2**·**31**, **26**·**05**	**6**·**47**	**1**·**83**, **22**·**87**
≥40 years	3·07	0·94, 10·02	**5**·**34**	**1**·**53**, **18**·**65**	**4**·**79**	**1**·**27**, **18**·**14**

PR, prevalence ratio; Ref. reference category. Model A shows adjusted relative PR from multinomial logistic regression that included sex and age at first migration. Model B is equal to Model A adjusted also by deprivation index, education level, smoking status, physical activity and alcohol consumption. Significant associations are shown in bold font.

## Discussion

Our results confirmed a trend of an increase of obesity prevalence according to the number of years of residence in urban areas among Peruvian rural-to-urban migrants. The relationship became stronger when adjusted for sex, age at first migration and other important confounding factors, such as deprivation index, education level, physical activity, smoking status and alcohol consumption.

On sensitivity analyses, this relationship was not observed in non-migrant groups, thus indicating that the effect observed can be ascribed to the migration experience. We also showed that migrant groups living in an urban area for more than 30 years have a 39 % higher prevalence of obesity when compared with migrants living in an urban area for less than 20 years. In our analysis of secondary outcomes by BMI, prevalence of obesity was much higher in those with longer years of urban residence. The relevance of this characterization of migration profiles relies on informing the design and targeting of obesity prevention interventions in similar groups.

Increased obesity risk in migrants compared with non-migrants, whether rural or urban populations, might lie in two important factors: rapid weight gain and acculturation. Childhood malnutrition is higher in deprived settings like rural areas or indigenous communities; this lack of nutrition during early periods of life is often followed by a rapid weight gain which is associated with obesity later in life^(^ ^29^ ^,^ ^30^ ^)^. Additionally, urban areas offer obesogenic conditions (i.e. highly energy-dense foods or sedentary lifestyles) that can impact dietary patterns of migrant populations through the process of acculturation^(^ ^31^ ^–^ ^33^ ^)^. Obesogenic conditions may accelerate weight gain during childhood and may increase the chances of obesity in adult populations proportionally with the length of urban residence.

The positive trend of an increase of obesity shown in migrants residing in urban areas for longer periods is consistent with the results of obesity risk in other studies^(^ ^15^ ^,^ ^18^ ^,^ ^19^ ^,^ ^34^ ^,^ ^35^ ^)^. The risk for obesity has been shown in different settings for rural-to-urban migrants^(^ ^34^ ^–^ ^39^ ^)^, as well as for international migrants moving to the USA^(^ ^14^ ^,^ ^15^ ^,^ ^40^ ^)^ and Portugal^(^ ^41^ ^)^. However, the magnitude of association reported varies among these studies and this issue might be related to the study design and methods of ascertainment of obesity. For instance, some studies used self-reported weight and height to calculate BMI^(^ ^14^ ^,^ ^16^ ^,^ ^42^ ^)^, while others objectively measured weight and height^(^ ^37^ ^,^ ^41^ ^)^.

In using the sum of four skinfolds and the Siri age- and sex-specific equation to calculate the percentage of body fat mass, we added a more sensitive measurement of obesity^(^ ^22^ ^,^ ^43^ ^,^ ^44^ ^)^ since obesity has been defined by the WHO as the excess of fat in the human body^(^ ^45^ ^)^. In previous reports of the PERU MIGRANT Study^(^ ^5^ ^,^ ^46^ ^)^, using BMI only, the prevalence of obesity and overweight in the rural-to-urban migrant group was reported at 21 % and 46 %, respectively. However, our study showed a prevalence of obesity of 78 % for the same group. Discrepancies in obesity prevalence calculated from BMI and skinfold measurements have been reported also by Minghelli *et al*., who found a threefold increase in the prevalence of obesity using the skinfold method compared with the BMI results^(^ ^47^ ^)^. This was also evident in our classification of participants, as nearly half of those with normal BMI status were indeed classified as obese based on skinfold measurements. Furthermore, secondary analysis of overweight and obesity by BMI categories showed similar results to our main analysis. While overweight prevalence did not differ by length of urban residence groups, obesity prevalence by BMI was greatly different in all the groups compared with the reference group. These results reconfirm the heterogeneity of addressing obesity using different anthropometric techniques. In reality, for wider public health and obesity prevention efforts, our results signal to the potential to reach different magnitudes of effect in epidemiological associations.

A potential explanation for these discrepancies lies with BMI limitations, which have been related to both differential and non-differential misclassification errors regarding body fat percentage that can produce bias, even more if the BMI is based on self-reported weight and height^(^ ^48^ ^)^. BMI does not disentangle the effect of fat mass, or adiposity, from lean mass since it takes whole body mass in the nutritional assessment^(^ ^49^ ^,^ ^50^ ^)^. Furthermore, BMI is dependent on age, sex^(^ ^51^ ^)^ and ethnicity^(^ ^52^ ^)^ when related to body fat mass or adiposity, which can lead to the paradox of low BMI and excess of body fat mass^(^ ^53^ ^,^ ^54^ ^)^. In our study, we found that almost half of the participants classified as normal by BMI status were classified as obese using skinfolds, which supports the statement that non-obese categories of BMI can hide high levels of adiposity or obesity^(^ ^55^ ^)^. Therefore, our study improves on the ascertainment of adiposity, taking advantage of skinfolds to characterize obesity through body fat mass. In so doing, our approach is better positioned to examine the relationship between within-country rural-to-urban migration and obesity.

Migrant studies have a challenge in disentangling the effects that length of urban residence and age at first migration have on different outcomes when age is present as a confounding factor because of the lack of independence between the latter and one of the first two^(^ ^18^ ^,^ ^56^ ^)^. Some studies exclude age as part of the final regression equation without explanation^(^ ^15^ ^,^ ^38^ ^)^, while in others the issue of multicollinearity is not assessed^(^ ^19^ ^)^. In our study, this lack of independence was shown through the strong correlation between age, length of urban residence and the sum of length of urban residence and age at first migration. Furthermore, our study found a high degree of multicollinearity between the three mentioned time-dependent variables: the mean VIF found in Model C was above 10 and even four times greater than the one reported in another migrant study about obesity risk in the USA^(^ ^18^ ^)^. In addition, we performed different analysis that confirmed this multicollinearity, such as correlation matrix of coefficients and the matrix of independent variables. After this detailed evaluation, it was decided to preserve Model B – the model including length of urban residence and age of first migration only – as the final multivariable regression model to be used. Despite these challenges, particularly in today’s world with ongoing patterns of human mobilization, migrants appear a suitable target group for obesity prevention initiatives^(^ ^57^ ^)^.

The present study shows scientific evidence that strengthens the relationship between urban residence and obesity in rural-to-urban migrants. First, the study has calculated obesity using four skinfold sites and the sex- and age-specific Siri equation that is a more specific index of adiposity than the BMI alone^(^ ^22^ ^,^ ^43^ ^)^. Furthermore, multicollinearity that is rarely assessed in migrant studies was evaluated and characterized in detail in the study; thus informing of potential explanations for non-significant associations between length of urban residence and obesity found in previous publications^(^ ^16^ ^,^ ^17^ ^,^ ^58^ ^,^ ^59^ ^)^. In addition, we had access to well-defined non-migrant groups, both in rural and in urban settings, that confirmed that the association of interest explored in the study was not explained by an age effect alone.

Some limitations in our study deserve consideration. Causality cannot be established because of the study’s cross-sectional nature; obesity, rapid weight gain or other risk factors could exist before migration. Yet, given the long-term exposure to urban environments, we could argue that migration precedes the development of obesity. Data from the PERU MIGRANT Study were collected in 2007 and obesity in rural areas has increased since then due to the nutritional transition; however, the increment from 2007 until 2011 was only 0·3 kg/m^2^ in the mean BMI in rural areas and differences with urban area still remained^(^ ^60^ ^)^. Skinfold methods have shown difficulty in measuring skinfolds precisely in adults with high levels of obesity^(^ ^61^ ^)^; consequently, each skinfold site was measured three times by trained professionals^(^ ^20^ ^)^. Reproducibility of results based on skinfold measurements is less than for other anthropometric measurements^(^ ^62^ ^)^; however, minimum technical errors and coefficient variation can be achieved as shown in the HERITAGE Family Study^(^ ^63^ ^)^. Although the Durnin and Wormsley equation has been recommended for Hispanic groups^(^ ^22^ ^)^ and has been used as reference method for the construction of new prediction equations in the Chilean population^(^ ^64^ ^)^, it is important to highlight that racial differences in body composition can affect the precision of the estimates of body fat mass from prediction equations^(^ ^65^ ^)^. Length of urban residence can serve as an indicator of acculturation^(^ ^66^ ^)^ and might have an effect on lifestyles and dietary changes^(^ ^2^ ^,^ ^66^ ^,^ ^67^ ^)^ which can increase the risk of obesity. Dietary information was not collected in the PERU MIGRANT Study; yet, given the long-term nature of our exposure–outcome association of interest, we anticipated that short-term dietary recall instruments could also have limitations. Furthermore, the migration patterns observed did not allow for more detailed assessments of shorter exposures to urban residency, i.e. a better characterization of the <20 years, used as reference group, which could certainly affect the magnitude of associations observed in our study. Last but not least, despite the effect of multicollinearity between age and length of residence, our regression Model B is not exempt from the residual effect of age in the hypothesized association.

## Conclusion

Length of urban residence affects the health of rural-to-urban migrant populations in Peru, by increasing their obesity risk in accordance with the number of years living in urban areas. Therefore, rural-to-urban migrant populations should be targeted for nutritional interventions in order to avoid the increase of the obesity rate and its effects on health outcomes in Peru.

## Supplementary material

For supplementary material accompanying this paper visit http://dx.doi.org/10.1017/S1368980015002578. click here to view supplementary material
