# Supplementary material for: Length of urban residence and obesity among within-country rural-to-urban Andean migrants
Source: Public Health Nutr. 2015 Sep 14;19(7):1270–8. doi: 10.1017/S1368980015002578 (PMC4825096; doi:10.1017/S1368980015002578)
Supplement: Supplementary file 1 [file S1368980015002578sup.zip › S1368980015002578sup001.doc]

## Supplemental Fig. 1: Graph matrix of the relation between years lived in urban area, age and age at first migration.

**R=0.74**

**R=-0.23**

**R=0.33**
